# Characterization of Two Small Heat Shock Protein Genes (*Hsp17.4* and *Hs20.3*) from *Sitodiplosis mosellana*, and Their Expression Regulation during Diapause

**DOI:** 10.3390/insects12020119

**Published:** 2021-01-29

**Authors:** Jiajia Zhao, Qitong Huang, Guojun Zhang, Keyan Zhu-Salzman, Weining Cheng

**Affiliations:** 1 Key Laboratory of Plant Protection Resources & Pest Management of the Ministry of Education, College of Plant Protection, Northwest A&F University, Yangling 712100, China; b20193190927@cau.edu.cn (J.Z.); 13760637249hqt@nwafu.edu.cn (Q.H.); zhangguojun97@nwafu.edu.cn (G.Z.); 2 Department of Entomology, Texas A&M University, College Station, TX 77843, USA

**Keywords:** *Sitodiplosis mosellana*, *Hsp17.4*, *Hsp20.3*, diapause, stress tolerance, ecdysone

## Abstract

**Simple Summary:**

Small heat shock proteins (sHsps) play important roles in thermal adaptation of various organisms, and insect diapause. *Sitodiplosis mosellana*, a key pest of wheat worldwide, undergoes obligatory larval diapause in soil to survive adverse temperature extremes during hot summers and cold winters. The objectives of this study were to characterize two *sHsp* genes from *S. mosellana* (*SmHsp17.4* and *SmHsp20.3*), and determine their expression in response to diapause, extreme high/low temperatures, or 20-hydroxyecdysone (20E) treatment. Expression of *SmHsp17.4* was down-regulated upon entry into diapause, but up-regulated during the shift to post-diapause quiescence. In contrast, expression of SmHsp20.3 was not affected by entry into diapause, but was pronounced during summer and winter. Furthermore, transcripts of both *SmHsps* were highly responsive to heat (≥35 °C) and cold (≤−5 °C) during diapause, and topical application of 20E on diapausing larvae also induced *SmHsp17.4* in a dose-dependent manner. Notably, the recombinant SmHsp17.4 and SmHsp20.3 exhibited significant molecular chaperone activity. In conclusion, *SmHsp17.4* and *SmHsp20.3* play essential roles in heat/cold adaptation, and 20E-mediated *SmHsp17.4* was also likely involved in diapause termination. Results have improved our understanding of the molecular mechanism underlying diapause and related stress tolerance in *S. mosellana*.

**Abstract:**

*Sitodiplosis mosellana*, a periodic but devastating wheat pest that escapes temperature extremes in summer and winter by undergoing obligatory diapause. To determine the roles of small heat shock proteins (sHsps) in diapause of *S. mosellana,* we characterized two *sHsp* genes, *SmHsp17.4* and *SmHsp20.3*, from this species. Both SmHsps contained the conserved α-crystallin domain and the carboxy-terminal I/VXI/V motif of the sHsp family. *SmHsp17.4* had one intron while *SmHsp20.3* had none. Quantitative PCR revealed that *SmHsp17.4* expression decreased after diapause initiation, but substantially increased during transition to post-diapause quiescence. In contrast, *SmHsp20.3* expression was not affected by entry of diapause, but was clearly up-regulated during summer and winter. Short-term more severe heat-stress (≥35 °C) of over-summering larvae or cold-stress (≤−5 °C) of over-wintering larvae could stimulate higher expression of both genes, and *SmHsp17.4* was more responsive to cold stress while *SmHsp20.3* was more sensitive to heat stress. Notably, transcription of *SmHsp17.4*, but not *SmHsp20.3*, in diapausing larvae was inducible by 20-hydroxyecdysone (20E). Recombinant SmHsp17.4 and SmHsp20.3 proteins also displayed significant chaperone functionality. These findings suggest that both *SmHsps* play key roles in stress tolerance during diapause; and 20E-regulated *SmHsp17.4* was also likely involved in diapause termination.

## 1. Introduction

In response to various environmental stresses such as extreme temperatures, heavy metals, pesticides, UV-A light, and anoxia, almost all organisms synthesize a set of proteins called heat shock proteins (Hsps) [1,2,3,4,5]. These molecular chaperones, up-regulated during stresses, promote correct refolding and thus prevent irreversible aggregation of denatured proteins. They are traditionally divided into several families based on molecular weight, including Hsp100, Hsp90, Hsp70, Hsp60, Hsp40, and small Hsps (sHsps) [6,7].

sHsps are ATP-independent proteins with molecular weights ranging from 12 to 43 kDa [8,9]. Compared to other Hsps, these proteins generally exhibit a greater variation in size, sequence, and function [10,11]. Ten sHsps were identified from the human genome and more than 10 sHsps were found in genomes of *Bombyx mori*, *Drosophila melanogaster,* and *Tribolium castaneum* [12,13]. They all possess a conserved α-crystallin domain containing 80–100 amino acid residues despite variable N- and C-terminal sequences. The α-crystallin domain, rich in β-strands, is thought to be crucial for maintaining chaperone activity of sHsps [14]. Apart from the primary chaperone function, sHsps participate in diverse fundamental cellular processes, involving cell growth, differentiation and apoptosis [15], membrane fluidity [16], and cytoskeletal organization [17,18]. They are also therapeutic targets and biomarkers of many human diseases [19,20].

Recent studies have shown that sHsps in insects are also involved in diapause, an adaptive strategy that insects develop to survive seasonal adverse environmental conditions such as temperature extremes [21,22]. For instance, in the flesh fly *Sarcophaga crassipalpis*, transcripts of *Hsp23* are most abundant in diapausing pupae, and decline when diapause ends and post-diapause development begins [23,24]. Up-regulation of *sHsps* during diapause have also been observed in several other insects, such as *Delia antiqua Hsp23* [25], *Calliphora vicina Hsps23* and *24* [26], *Helicoverpa armigera Hsp21.4*, and *Grapholita molesta Hsps18.9*, *21.3*, *21.7* and *31.8.* In contrast, *H. armigera Hsp20.7*, *G. molesta Hsp21.4*, and *Sesamia nonagrioides Hsp20.8* are down-regulated during diapause [27,28,29], and some other *sHsps* are not affected by diapause [30,31,32]. Thus roles played by sHsps in diapause might differ greatly amongst protein family members and insect species.

It has been suggested that functions of Hsps are regulated by ecdysone. Several *sHsp* genes, such as *D. melanogaster Hsps23* and *27* [33,34], *Ceratitis capitata Hsp27* [35], and *Apis cerana Hsps23* and *24.2* [36], are up-regulated by 20-hydroxyecdysone (20E) during normal development. The ecdysone-responsive element has been found in the promoter region of *C. capitata Hsp27* [35]. However, little information is available on the 20E regulation of *sHsps* during diapause.

The orange wheat blossom midge *Sitodiplosis mosellana* (Géhin) (Diptera: Cecidomyiidae), a widespread periodic wheat pest across Asia, Europe, and North America, causes severe damage in years of high infestation [37,38,39]. It is a univoltine species and undergoes an obligatory larval diapause in soil. Adults emerge and lay eggs in late April in most of northern China [40]. Hatched larvae feed on developing seeds. Mature 3rd instar larvae drop from wheat ears to the ground during middle to late May, and burrow into soil to form round diapausing cocoons. They do not exit cocoons until the mid-March of the following year. Therefore, the long period of diapause enables this insect to escape unfavorable temperature extremes during hot summers and cold winters. To date, sHsps have been widely considered to be the most crucial players in heat/cold adaptation and insect diapause, although the function of sHsps in diapausing *S. mosellana* remains to be investigated.

In the present study, we cloned and characterized two *sHsp* genes from *S. mosellana* (*SmHsp17.4* and *SmHsp20.3*). We profiled their expression during diapause and examined how excess short-term extreme temperatures or 20E treatment impacted expression patterns. In addition, we explored the potential chaperone activities of the recombinant proteins in vitro.

## 2. Materials and Methods

### 2.1. Experimental Insects

Pre-diapausing 3rd instar larvae of *S. mosellana* were collected from severely infested wheat ears from a wheat field on 20 May 2016. Wheat ears containing pre-diapausing 3rd instar larvae were also harvested in large quantity and put on moist soil in a field insectary in Northwest A&F University, Yangling (34°16′ N, 108°4′ E), Shaanxi, China, to facilitate larvae to enter diapause. Successful diapause initiation is indicated by formation of larval cocoons. Our earlier study showed that almost all cocooned larvae collected in December or later have terminated diapause and entered post-diapause quiescence [41]. Cocooned larvae at diapausing and quiescent stages, and post-diapause developing larvae (i.e., larvae exiting cocoons) were successively collected from soil in the insectary following the procedure we previously developed [42]. All larvae collected were frozen in liquid nitrogen and then stored at −80 °C for later RNA extraction.

### 2.2. RNA Extraction, cDNA Synthesis, and gDNA Isolation

Total RNA was extracted from 20 pre-diapausing larvae using the TRNizol RNA Isolation Kit (TaKaRa, Dalian, China). The concentration and quality of RNA was verified using a spectrophotometer and 1% agarose gel electrophoresis, respectively. Reverse transcription was performed with 1 μg of total RNA using the PrimeScript^TM^ RT Reagent Kit with gDNA Eraser (Perfect Real Time) (TaKaRa, Dalian, China). Genomic DNA was isolated from 20 pre-diapausing larvae with the Biospin Insect Genomic DNA Extraction Kit according to the manufacturer’s instructions (Bioer Technology Co., Ltd., Hangzhou, China).

### 2.3. Cloning of SmHsp17.4 and SmHsp20.3

Based on annotated *Hsp17.4* and *Hsp20.3* sequences from the transcriptome database of *S. mosellana* larvae, gene-specific primers (Table 1) were designed to amplify cDNA end sequences with 3’-Full RACE (rapid amplification of cDNA ends) Core Set with PrimeScript™ RTase and 5′-Full RACE Kit with TAP (TaKaRa, Dalian, China). Nested PCRs were conducted with the following program: 94 °C for 3 min, followed by four cycles of 94 °C for 30 s, 60 °C–58 °C for 30 s, and 72 °C for 90 s with a decrease of annealing temperature by 2 °C per two cycles; then 32 cycles of 94 °C for 30 s, 56 °C for 30 s, 72 °C for 90 s, and 72 °C for 10 min. PCR products were confirmed by 1% agarose gel, purified using a gel extraction kit (Tiangen, Beijing, China), ligated into pMD^TM^-19T vector (TaKaRa, Dalian, China), and then transformed into *Escherichia coli* DH5α-competent cells (Tiangen, Beijing, China) for sequencing analysis. Full-length cDNAs were subsequently PCR amplified using primers shown in Table 1 and Taq MasterMix (CWBio, Beijing, China).The amplification conditions were as follows: 95 °C for 3 min, followed by 4 cycles at 95 °C for 40 s, 56–54 °C for 50 s, and 72 °C for 2 min with a decrease of annealing temperature by 2 °C per two cycles; then 32 cycles at 95 °C for 40 s, 52 °C for 50 s, 72 °C for 2 min, and 72 °C for 10 min. PCR products were gel-purified, cloned, and sequenced. Genomic DNAs for *Hsp17.4* and *Hsp20.3* were also obtained by PCR using total DNA as the template and the sequence confirmed as above.

### 2.4. Sequence Analysis and Phylogenetic Tree Construction

The open reading frame (ORF) was analyzed using ORF finder (http://www.ncbi.nlm.nih.gov/gorf/gorf.html). Protein molecular weight and isoelectric point were deduced using the Compute pI/Mw tool (http://web.expasy.org/protparam/). Functional domains were identified using a conserved domain search tool (http://www.ncbi.nlm.nih.gov/Structure/cdd/wrpsb.cgi). Multiple sequence alignment was performed using the DNAMAN software (Lynnon Corporation, Pointe-Claire, QC, Canada). Secondary and three-dimensional (3D) structures were predicted using ESPrigt (http://espript.ibcp.fr/ESPript/cgi-bin/ESPript.cgi) and SWISS-MODEL Server (https://www.swissmodel.expasy.org/), respectively. Images were visualized using the PyMOL Molecular Graphics System (Version 1.3, Schrödinger, LLC, New York, NY, USA). The GMQE (Global Model Quality Estimation) and QMEAN (Qualitative Model Energy Analysis) values of the homology model were, respectively, 0.75 and −0.59 for SmHsp17.4, and 0.79 and 0.42 for SmHsp20.3. The phylogenetic tree was constructed using the neighbor-joining method in MEGA 6.0 software [43], and parameters included complete deletion, 1000 replicates for bootstrap analysis, and p-distance model.

### 2.5. Heat/Cold Shock Treatments

*S. mosellana* cocooned larvae are mainly located 3–10 cm below the soil surface [44]. Temperatures in this soil layer are around 32 °C (highest) in summer and around 0 °C (lowest) in winter in Yangling (34°16′ N, 108°4′ E), Shaanxi province, China. However, agricultural practices like tillage could occasionally expose the diapausing insects to extreme temperatures as high as 50 °C in summers or as low as −15 °C in winters. To elucidate the potential function of *SmHsp17.4* and *SmHsp20.3* under such conditions, over-summering and over-wintering larvae were further treated with heat or cold shock. Briefly, freshly cocooned larvae collected in August were put into 1.5 mL cryogenic vials, and then submerged in water bathes at various high temperatures (35–50 °C) for 1 h, and at 35 °C for various time periods (0–120 min). Samples from a 60 min heat shock at 35 °C were also subjected to a different recovery time (30–360 min) at room temperature. Similarly, vials containing December-cocooned larvae were placed in incubators with temperature controlled at various low temperature (0–15 °C) for 1 h, and at −10 °C for various time periods (0–120 min). Samples from a 60 min cold shock at −10 °C also experienced a different recovery time (30–360 min) at room temperature. Larvae collected prior to temperature treatments acted as the untreated control. After treatment at each time point, larvae were frozen immediately in liquid nitrogen and stored at −80 °C for reverse-transcription quantitative PCR (RT-qPCR) analysis. Each treatment was conducted at least three times with 20 individuals per replicate.

### 2.6. 20E Treatment

To ascertain whether 20-hydroxyecdysone (20E) affected expression of *SmHsp17.4* and *SmHsp20.3* in diapausing larvae, cocooned larvae freshly collected in October were abdomen-injected with 23 nL 20E (Sigma-Aldrich, St. Louis, MO, USA) at concentrations of 0.1–0.4 pg/nL diluted in 50% ethanol using a Nanoject II Auto-Nanoliter Injector (Drummond Scientific Company, Broomall, PA, USA). Insects at this stage were selected because they showed low expression of *SmHsp17.4* and *SmHsp20.3* (see Results). Injection doses were comparable to endogenous 20E levels reported in larvae of this species [45]. Injected larvae were placed in petri dishes with moist filter papers and continuously incubated for 3, 6, or 12 h at 25 °C in the artificial climate incubator prior to being frozen in liquid nitrogen and stored in a −80 °C freezer for RNA isolation later on. Control insects were treated with the same volume of 50% ethanol. Each treatment was repeated at least three times with a minimum of 20 individuals per replicate.

### 2.7. Expression Analysis Using RT-qPCR

To determine the expression profile of *SmHsp17.4* and *SmHsp20.3* in response to diapause, extreme heat/cold stresses, and 20E treatment, total RNA of each sample was isolated and cDNAs were synthesized from 1.0 μg total RNA as described above. Three replicates were conducted for each sample.

RT-qPCR was performed using the SYBR Premix EX Taq^TM^ II kit (Tli RNaseH Plus) (Takara, Dalian, China) on the QuantStudio^®^5 real-time PCR system (Thermo Fisher Scientific, Waltham, MA, USA) under the following conditions: 95 °C for 30 s, then 40 cycles of 95 °C for 5 s, 60 °C for 30 s, and 72 °C for 30 s. The reference gene gapdh (GenBank number: KR733066) was used as the experimental control. The result showed that the amplification efficiencies for *gapdh*, *SmHsp17.4*, and *SmHsp20.3* were respectively 103%, 96%, and 97%, which were automatically calculated by a real-time PCR system. The transcript abundance of each *SmHsp* gene (for primers, see Table 1) was normalized to that of the reference gene, and the relative expression was calculated with the 2^−ΔΔCT^ method [46]. Data were shown as means ± SE (standard error). Differences among treatments was analyzed by one-way analysis of variance followed by Duncan’s multiple range tests (*p* < 0.05). The relationship between 20E levels and *sHsp* expression was analyzed using correlation analysis.

### 2.8. Recombinant Protein Expression and Purification

To construct *SmHsp17.4* and *SmHsp20.3* expression vectors, specific primers with restriction sites designed at their 5′ ends (BamHI in the sense primer and HindIII in the anti-sense primer) were used to amplify the coding sequences of *SmHsp17.4* and *SmHsp20.3* (Table 1). The products, confirmed by sequencing, were digested and inserted into the linearized pET28a (+) expression vector (Novagen, Madison, WI, USA) restricted by the same restriction enzymes. The construct containing *SmHsp17.4* gene was transformed into *E. coli* Rosetta cells, whereas the *SmHsp20.3* construct was transformed into *E. coli* BL21 cells (Tiangen, Beijing, China). A single recombinant colony was grown overnight at 37 °C in 5 mL of Luria-Bertani (LB) medium with 100 μg/mL kanamycin. The culture was then diluted 100-fold with fresh LB medium (500 mL) and continuously incubated at 37 °C until the optical density at 600 nm (OD_600_) reached 0.6–0.8, when isopropyl β-d-1-thiogalactopyranoside (IPTG) was added to a final concentration of 0.5 mM, followed by a further incubation with shaking for 5 h at 37 °C. Cells were harvested by centrifugation at 8000× *g* for 10 min, re-suspended in the lysis buffer (20 mM Tris-HCl pH 7.4, 0.5 mM phenylmethanesulfonyl fluoride), lysed using 0.4 mg/mL lysozyme and repeated freeze–thaw cycles in liquid nitrogen, and then centrifuged again at 12,000× *g* for 30 min. Recombinant protein expression was examined in supernatant and pellet with 15% sodium dodecyl sulfate polyacrylamide gel electrophoresis (SDS-PAGE).

Insoluble SmHsp17.4 was denatured with 8 M urea and refolded by following a previously-described procedure [47]. The solubilized SmHsp17.4 and soluble SmHsp20.3 were purified using the Ni-NTA His·Bind Resin column (7sea Pharmatech Co., Shanghai, China), and examined by 15% SDS-PAGE.

### 2.9. Thermal Aggregation Assays

To determine if recombinant SmHsp17.4 and SmHsp20.3 could suppress thermal aggregation of pig heart mitochondrial malate dehydrogenase (MDH), 0.5 μM MDH in PBS buffer was incubated at 43 °C in an Infinite M1000 PRO microplate reader (Tecan Group Ltd., Männedorf, Switzerland), alone or in the presence of SmHsp17.4, SmHsp20.3, bovine serum albumin (BSA, the non-chaperone control) in a molar ratio of 1:1. Absorbance at 360 nm was measured every 10 min for 70 min [48]. Three replications were conducted for each sample.

## 3. Results

### 3.1. Characterization of SmHsp17.4 and SmHsp20.3

The full-length cDNA of *S. mosellana Hsp17.4* (*SmHsp17.4*) obtained by RACE-PCR was 773-bp (Figure S1, GenBank accession no: MK714040), which contained a 144-bp 5′ untranslated region (UTR), a 456-bp open reading frame (ORF), and a 173-bp 3′UTR. The predicted ORF encoded a protein of 151 amino acids with an estimated molecular weight of 17.4 kDa and an isoelectric point (pI) of 6.87. The cDNA sequence of *SmHsp20.3* was 803 bp long (Figure S1, GenBank accession no: MK714041), and contained a 81-bp 5′ UTR, a 543-bp ORF, and a 179-bp 3′ UTR. The ORF encoded a protein of 180 amino acids with molecular weight of 20.3 kDa and a pI of 5.80. Comparison between cDNA and gDNA sequences revealed that *SmHsp17.4* contained one 85 bp intron at position 108 bp downstream of start codon ATG (Figure S2), while SmHsp20.3 had none.

**Figure 1:**
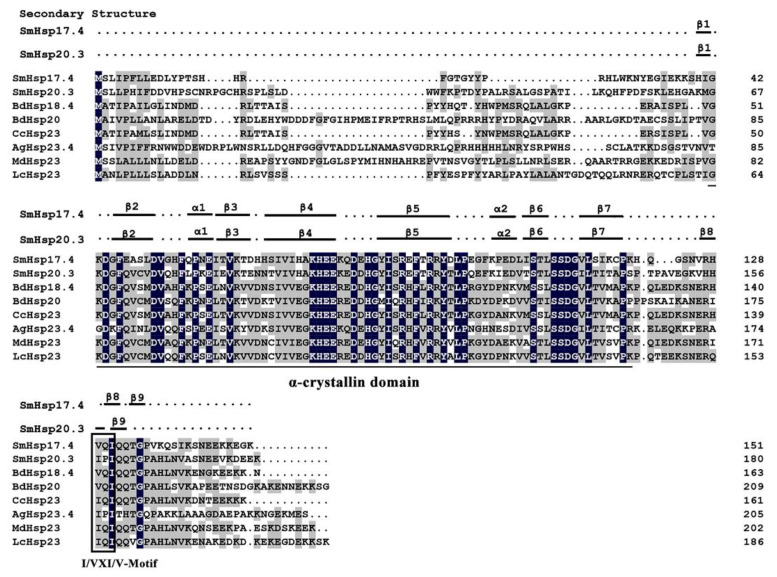
Amino acid sequence alignment of SmHsp17.4, SmHsp20.3, and *s*mall heat shock proteins (sHsps) from other insect species. Identical and similar residues are shaded black and grey, respectively. The α-crystallin domain is underlined and the I/VXI/V motif is boxed. Putative secondary structures are labeled above sequences as α (α-helices), β (β-strands), and dotted lines (coils), respectively. Sm, *Sitodiplosis mosellana*; Bd, *Bactrocera dorsalis*; Cc, *Ceratitis capitata*; Ag, *Anopheles gambiae*; Md, *Musca domestica*; and Lc, *Lucilia cuprina*.

Searching for conserved domains identified the α-crystallin domain in both SmHsps—79 amino acid residues (42–120) in SmHsp17.4 and 78 residues (67–144) in SmHsp20.3. The I/VXI/V motif was also present in the C-terminal regions (a.a. 129–131 in SmHsp17.4 and a.a. 157–159 in SmHsp20.3) (Figure 1 and Figure S1). In addition, 21 glutamine and glutamic acid residues, known to promote protein stability under elevated temperatures by providing additional electrostatic force [49], were detected. Only one cysteine residue (C-118) was found in SmHsp17.4 and three in SmHsp20.3 (C-15, C-20, and C-74), consistent with the study of Fu et al. (2003) [50] who concluded that cysteine residues were rare in the sequences of molecular chaperones compared to other protein families.

**Figure 2:**
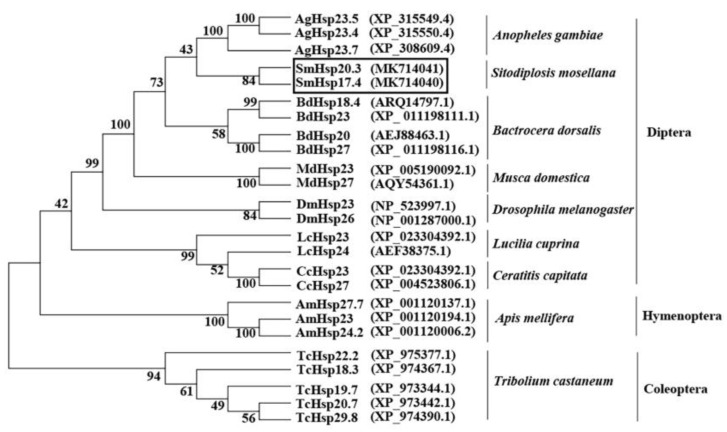
Phylogenetic relationship of SmHsp17.4 and SmHsp20.3 (boxed) as well as sHsps from other insects. The phylogenetic tree was built by the neighbor-joining method using MEGA 6.0 with 1000 bootstrap replications.

Sequence alignment demonstrated that SmHsp17.4 exhibited the highest amino acid sequence identity (57%) to *Bactrocera dorsalis* Hsp18.4 (ARQ14797.1), 49–55% identity to *B. dorsalis* Hsp20 (AEJ88463.1), *Anopheles gambiae* Hsp23.4 (XP_315550.4), and Hsp23 from *Ceratitis capitate* (XP_004523813.1), *Musca domestica* (XP_005190092.1), and *Lucilia cuprina* (XP_023304392.1). SmHsp20.3 was 59% identical to Hsp23 from *C. capitate* and *L. cuprina*, and 52–56% identical to *B. dorsalis* Hsp18.4, *B. dorsalis* Hsp20, *A. gambiae* Hsp23.4, and *M. domestica* Hsp23. The two SmHsps were 43% identical (Figure 1). The phylogenetic analysis revealed that different sHsps of the same insect species tended to cluster together, and the two SmHsps were most closely related to sHsps from the Nematocera (*A. gambiae)* in Diptera (Figure 2).

**Figure 3:**
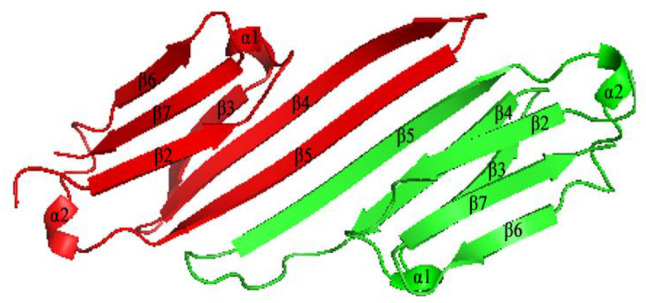
Predicted α-crystallin domain dimeric structure of SmHsp17.4 and SmHsp20.3. The SWISS MODEL server was used to simulate three-dimensional structure based on the defined zebrafish (*Danio rerio*) homolog (PDB ID: 3n3e.1A). Image was visualized using PyMOL software. Individual monomers are marked red and green.

Analysis of the secondary structure revealed six β-strands and two α-helixes in the α-crystallin domain of the SmHsps (Figure 1). The predicted three-dimensional structure was generated based on the zebrafish (*Danio rerio*) homolog (PDB ID: 3n3e.1A), presumably the best template as it shared 47.4% and 49.4% sequence identity with SmHsp17.4 and SmHsp20.3, respectively. For both SmHsps, the conserved α-crystallin domain existed in a dimeric structure. In each monomer, six β-strands were folded into a compact β-sandwich composed of two antiparallel β-sheets (Figure 3).

### 3.2. Expression of SmHsp17.4 and SmHsp20.3 during Diapause

**Figure 4:**
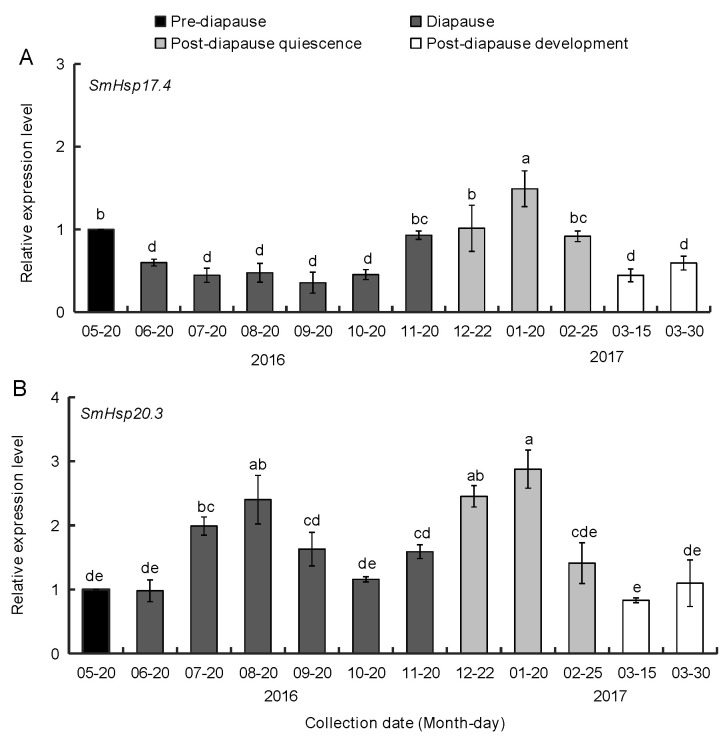
Expression profiles of *SmHsp17.4* (**A**) and SmHsp20.3 (**B**) in pre-diapausing, diapausing, and post-diapausing larvae by RT-qPCR. The expression level of each tested stage was relative to that of the pre-diapause stage, which was arbitrarily set at 1. Bars represent the means ± SE. Different letters above bars indicated significant difference by Duncan’s multiple range test (*p* < 0.05).

Transcript profiles of *SmHsp17.4* and *SmHsp20.3* during diapause, including pre-diapause (May), diapause (June–November), post-diapause quiescence (December–next February), and post-diapause development (March of the next year) were determined by RT-qPCR. Expression of *SmHsp17.4* decreased after the initiation of diapause (June), stayed low during diapause maintenance (June to October), but significantly increased in November, a transition time from diapause to post-diapause quiescence, and peaked in December and January. Expression distinctly decreased thereafter, and returned to the early-diapause level after development resumed (Figure 4A). In contrast, expression of *SmHsp20.3* did not change when *S. mosellana* entered diapause (June), but peaked in July and August and the early-to-mid period of post-diapause quiescence (December and January) (Figure 4B). Expression was relatively low at the post-diapause developmental stage.

### 3.3. Effect of Temperature Extremes on SmHsp Expression during Diapause

**Figure 5:**
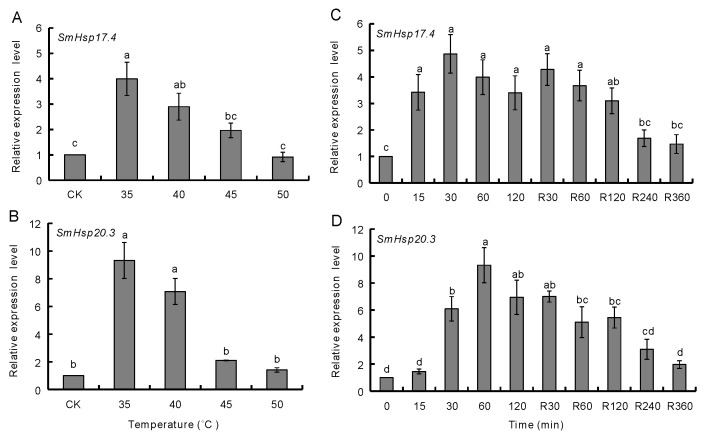
Effect of high temperature extremes on expression of *SmHsp17.4* and *SmHsp20.3* in over-summering diapausing larvae. (**A**,**B**) Exposure to various high temperature (35–50 °C) for 1 h. (**C**,**D**) Exposure to 35 °C for various time periods (0–120 min, left side), as well as a 60 min exposure to 35 °C followed by different recovery time (R30–360 min, right side). Expression level of each treatment was relative to that of the untreated control (CK, 0 min), which was arbitrarily set at 1. Bars represent the means ± SE. The different letters above the columns indicate significant difference by Duncan’s multiple range test (*p* < 0.05).

When over-summering diapausing larvae were treated with various temperatures higher than the typical habitat temperature in the hottest period of the summer for 60 min, expression of *SmHsp17.4* and *SmHsp20.3* was significantly higher at the temperature ranging from 35 to 40 °C than the untreated control; a 4.0-fold increase for *SmHsp17.4* and a 9.4-fold for *SmHsp20.3* were observed at 35 °C, but such up-regulation was not seen in the 45 to 50 °C range (Figure 5A,B).

We also examined the response of both genes to heat shock duration at 35 °C as well as to recovery time at room temperature from 60 min exposure to 35 °C (Figure 5C,D). *SmHsp17.4* expression significantly increased upon 15 min of heat shock, remained at similar levels as treatment duration was extended (120 min) and during the first 60 min of recovery, before declining. Expression of *SmHsp20.3* significantly increased at 30 min of heat shock, peaked at 60 min, and gradually declined after 30 min of recovery. After recovering for 240 min, expression of both genes returned to the control levels.

**Figure 6:**
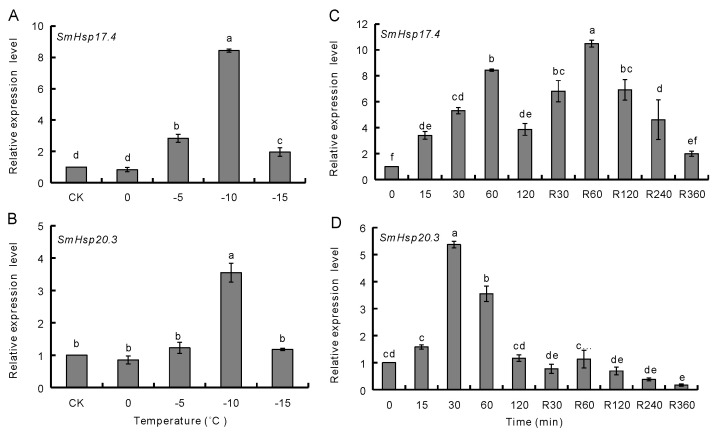
Effect of low temperature extremes on expression of SmHsp17.4 and SmHsp20.3 in over-wintering diapausing larvae. (**A**,**B**) Exposure to various low temperature (0–15 °C) for 1 h. (**C**,**D**), Exposure to −10 °C for various time periods (0–120 min, left side), as well as a 60 min exposure to −10 °C followed by different recovery time (R30–360 min, right side). Expression level of each treatment was relative to that of the untreated control (CK, 0 min), which was arbitrarily set at 1. Bars represent the means ± SE. The different letters above the columns indicated significant difference by Duncan’s multiple range test (*p* < 0.05).

Likewise, *SmHsp17.4* and *SmHsp20.3* expression were determined in over-wintering larvae subjected to further cold-shock treatments for 1 h at various low temperatures (Figure 6A,B). *SmHsp17.4* was significantly induced at −5 °C, −10 °C, and −15 °C, with the maximum expression level at −10 °C (approximately 8.4-fold). Up-regulation of *SmHsp20.3*, however, was observed only at −10 °C (3.6-fold).

Cold shock duration and different subsequent recovery time had different effects on the two genes (Figure 6C,D). When exposed to −10 °C, *SmHsp17.4* transcripts rapidly increased at 15 min, and reached maximum at 60 min. *SmHsp20.3*, however, responded at 30–60 min cold treatment, with the expression peaked at 30 min. During the recovery period following a 60 min exposure at −10 °C, *SmHsp17.4* remained high at the first 120 min and gradually dropped thereafter whereas *SmHsp20.3* rapidly decreased to the minimal level.

### 3.4. 20E Regulation of SmHsp17.4 and SmHsp20.3 during Diapause

**Figure 7:**
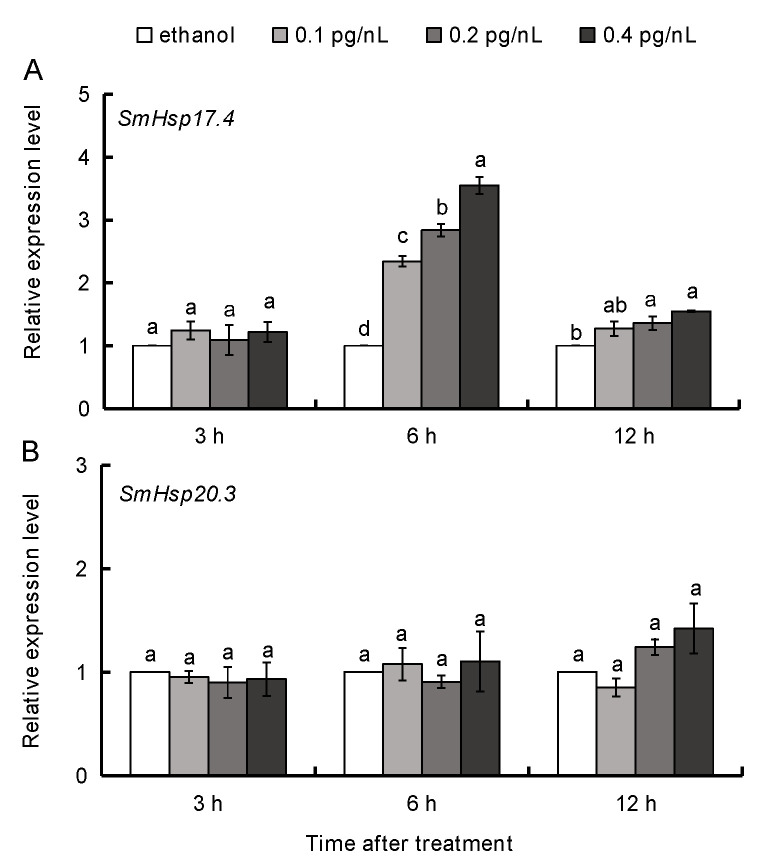
Effect of 20-hydroxyecdysone (20E) on expression of *SmHsp17.4* (**A**) and *SmHsp20.3* (**B**) in diapausing larvae collected in October. Transcript levels were determined by RT-qPCR at 3, 6, and 12 h after 20E (0–0.4 pg/nL) injection. Expression level of each treatment was relative to that of 50% ethanol injection (the control), which was arbitrarily set at 1. Bars represent the means ± SE. The different letters above the columns within each group indicated significant difference by Duncan’s multiple range test (*p* < 0.05).

Different expression patterns of two *SmHsp*s were observed when diapausing larvae (collected in October) were subjected to 20E treatment. *SmHsp20.3* did not change, while *SmHsp17.4* transcripts were greatly induced at 6 h post treatment, and showed a strong positive relationship with the 20E concentration (*r =* 0.932, *p* < 0.01). Some residual effect was also observed at 12 h after treatment (*r =* 0.835, *p* = 0.001). No notable change was observed 3 h after 20E injection at any concentration examined (Figure 7).

### 3.5. Chaperone Activity of Recombinant SmHsp17.4 and SmHsp20.3

**Figure 8:**
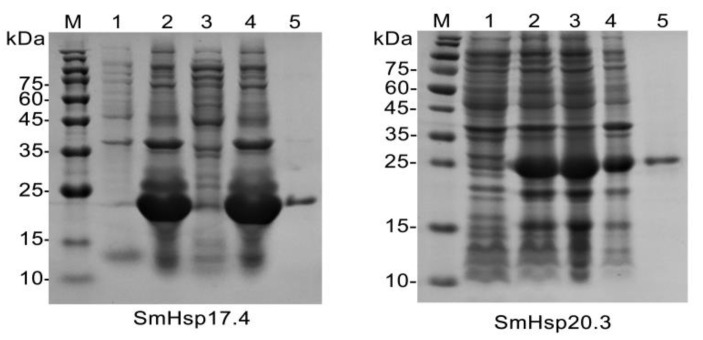
Expression and purification of recombinant SmHsp proteins by SDS-PAGE. M, molecular weight marker; lanes 1 and 2, un-induced and induced *E. coli* with pET28a (+)/SmHsps, respectively; lanes 3 and 4, supernatant and inclusion body of pET28a (+)/SmHsps after IPTG induction, respectively; lane 5, Ni-NTA affinity-purified pET28a (+)/SmHsp proteins.

After IPTG induction, SmHsp17.4 was present mainly in inclusion bodies whereas SmHsp20.3 was expressed as a soluble protein (Figure 8). Successful refolding and purification were demonstrated by SDS-PAGE (~22 kD and 25 kD, including His-tag, respectively). The concentrations of the purified proteins were 637 μg/mL for SmHsp17.4 and 84 μg/mL for SmHsp20.3.

**Figure 9:**
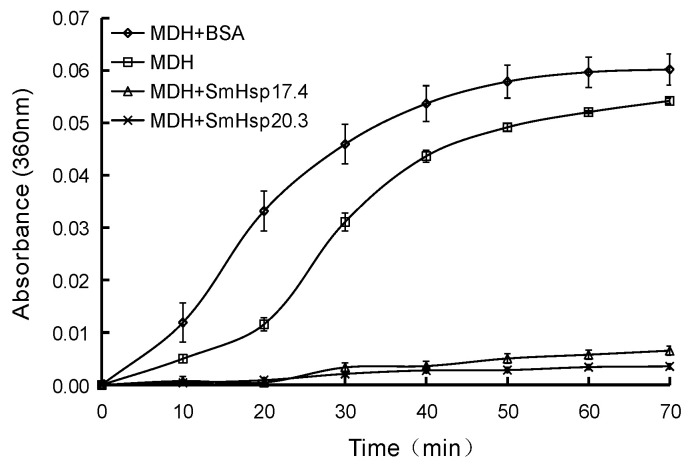
Molecular chaperone activity of SmHsp17.4 and SmHsp20.3. To perform mitochondrial malate dehydrogenase (MDH) thermal aggregation assays, MDH (0.5 μM) was incubated at 43˚C in the absence and in the presence of SmHsp17.4, SmHsp20.3, or bovine serum albumin (BSA, control) in a molar ratio 1:1. Aggregation of MDH was reflected by the absorbance at 360 nm. Data were obtained from three independent experiments and were shown as the means ± SE.

In the thermal aggregation assays (Figure 9), mitochondrial malate dehydrogenase (MDH) aggregated when the solution was incubated at 43 °C in the absence of the SmHsps, as indicated by the remarkable increase in absorbance at 360 nm. This heat-induced aggregation of MDH, however, did not occur in the presence of SmHsp17.4 or SmHsp20.3, suggesting their significant molecular chaperone functionality. The non-chaperone BSA control not only failed to prevent heat-induced aggregation, but sped up this process as well.

## 4. Discussion

After characterization of several high-molecular-weight heat shock proteins, Hsp90, Hsp70/Hsc70, and Hsp40 in *S. mosellana* [42,51], in the current study, we identified two members of the sHsps from this species, *SmHsp17.4* and *SmHsp20.3*. Sequence alignment revealed conserved motifs—α-crystallin domain that is essential for the molecular chaperone function, as well as the I/VXI/V motif that acts as an anchor in the oligomerization process [52]. Like most sHsps, α-crystallin domain of SmHsp17.4 and SmHsp20.3 is rich in β-strands organized into the β-sheet (Figure 1 and Figure 3), which is the basic structural unit for sHsps and helps facilitate dimer formation and subsequent oligomerization of sHsps [10,14,19]. Phylogenetic analysis suggested that different sHsps of the same species are clustered together. This is in agreement with a previous study covering a great number of sHsps from fungi, insects, plants, and vertebrates [53], suggesting that sHsps may have evolved by gene duplication after species divergence.

Given that a specific sHsp member may have a unique response and function in coping with stresses during insect diapause [24,25,26,27,28,29], we profiled expression of the newly-cloned *SmHsp* genes. We used the *S. mosellana* population that was exposed to natural conditions, rather than that raised under lab conditions, as in most molecular studies related to diapause. Our study with field-collected *S. mosellana* indicated that *SmHsp17.4* was down-regulated upon entry into diapause, but winter low temperature, a necessary factor to terminate diapause [41], greatly elevated expression (Figure 4A). Therefore, *SmHsp17.4* might be involved in the initiation and termination of diapause. Because *SmHsp17.4* was upregulated at an early stage in the transition from diapause to post-diapause quiescence, a stage morphologically indistinguishable from diapause, it could serve as a useful biomarker to predict diapause termination. Such a biomarker role has also been proposed for *S. nonagrioides Hsp20.8* [27] that displays a similar expression pattern to *SmHsp17.4* during diapause.

Larval diapause is known to be directly regulated by juvenile hormone and the steroid hormone ecdysone (mainly 20E) [54,55]. Interestingly, 20E titers in *S. mosellana* decline dramatically after entering diapause but significantly increase in December and January to levels higher than any other diapause periods [45]. Such a titer pattern coincides with the *SmHsp17.4* expression profile. Moreover, *SmHsp17.4* transcription in diapause larvae was inducible by 20E (Figure 7A), suggesting that ecdysone is responsible for transcriptional regulation of *SmHsp17.4* during this specific developmental stage. To our knowledge, this is the first report on 20E regulation of an sHsp gene during insect diapause despite many studies on such regulation during normal development [33,34,35,36].

*SmHsp20.3* expression was not affected by entry into diapause and 20E, but was clearly induced by seasonally-high and -low temperatures (Figure 4B and Figure 7B), implying that SmHsp20.3 is likely involved in heat/cold stresses during *S. mosellana* diapause, but is unrelated to hormone regulation. *SmHsp20.3* is intronless, and such sHsps are believed to be associated with responses to environmental stresses [2]. Conversely, sHsps with at least one intron, like *SmHsp17.4*, are mostly involved in metabolic processes [13]. Such an assumption may explain the higher induction of *SmHsp20.3* than *SmHsp17.4* by seasonal temperature changes.

More severely heat-stressed (≥35 °C) over-summering larvae or cold-stressed (≤−5 °C) over-wintering larvae rapidly elevated expression of the *SmHsp*s, but such induction did not last long (Figure 5 and Figure 6). It is possible that *SmHsp*s provide short protection when diapausing *S. mosellana* are exposed to thermal extremes on soil surface by agricultural practices like tillage [56,57]. Enhancing stress tolerance was also reflected by their chaperone activity evidenced by the thermal aggregation assays (Figure 9). Clearly, *SmHsp17.4* was more responsive to cold stress and *SmHsp20.3* was more sensitive to heat stress (Figure 4, Figure 5 and Figure 6), implying that *SmHsp17.4* may be more prominent for cold tolerance and *SmHsp20.3* for heat tolerance.

## 5. Conclusions

In summary, our results suggested that *SmHsp17.4* and *SmHsp20.3* genes may contribute to stress tolerance in *S. mosellana*, and that their higher expression in diapausing insects in hot or cold environments could protect *S. mosellana* from stress-induced injuries. Given its regulation by 20E, *SmHsp17.4* was also likely involved in the initiation and termination of the diapause process. Given that RNAi is not yet technically feasible for *S. mosellana*, technical improvement for in vivo functional analysis specific for this particular species is necessary to understand the molecular mechanism underlying diapause.

## Figures and Tables

**Table 1:** Primer sequences used in this study.

Primer Name	Sequence (5′ to 3′)	Purpose
Hsp17.4-5′-outer	CTTGTTTCTCTTCGTGTTTGGCATG	5′ RACE
Hsp17.4-5′-inner	GTGACGCTTCGAAACCATCCTTG	
Hsp20.3-5′-outer	GATCGCAAAGCTGGATAATCGGTTG	
Hsp20.3-5′-inner	CGTCAAAAATGTGTGGCAATAGAGAC	
Hsp17.4-3′-outer	CTACTCTCTCATCTGATGGTGTTCTCTC	3′ RACE
Hsp17.4-3′-inner	CAGAGCATCAAGAGCAACGAGGAG	
Hsp20.3-3′-outer	CAAACGGGACCAGCTCACTTGAATG	
Hsp20.3-3′-inner	AGCAACGAAGAAGTGAAAGACGAAG	
Hsp17.4-F1	AGTCGAATCTAAAGCATTCC	Full-length cDNA validation and gDNA cloning
Hsp17.4-R1	GGTCCTTTATATTGATTGAAATTTAC	
Hsp20.3-F1	TTATACGAATCGTTAACGAAAC	
Hsp20.3-R1	AATGAATTTCAAAATTCGCTCTTAG	
Hsp17.4-F2	CGGGATCCATGTCGTTGATTCCATTCC (BamHI)	Prokaryotic expression
Hsp17.4-R2	CCCAAGCTTGGGTTACTTTCCCTCTTTTTTC (HindIII)	
Hsp20.3-F2	CGGGATCCATGTCTCTATTGCCACACA (BamHI)	
Hsp20.3-R2	CCCAAGCTTGGGTTATTTTTCTTCGTCTTTC (HindIII)	
Hsp17.4-q-F	GAGCACGGTTACATTTCGC	qPCR
Hsp17.4-q-R	CTCCTCGTTGCTCTTGATGCTCTGTTTGACT	
Hsp20.3-q-F	AAGCCGTCCTTGCCCATTT	
Hsp20.3-q-R	ATTATCGCTTGACTGGTGG	
GAPDH-q-F	CCATCAAAGCAAGCAAGA	
GAPDH-q-R	CAGCACGGAGCACAAGAC	

Restriction sites are underlined.

## Data Availability

The data supporting reported results are available in the Supplementary Materials of this article.

## Supplementary Materials

The following are available online https://www.mdpi.com/2075-4450/12/2/119/s1: Figure S1: Nucleotide and deduced amino acid sequences of SmHsp17.4 and SmHsp20.3. Start codons (ATG) and stop codons (TAA) are boxed. The α-crystallin domain was shaded. I/VXI/V motif was indicated with oval. Figure S2: Genomic DNA sequence of SmHsp17.4. Start codon (ATG) and stop codon (TAA) were boxed. The intron was shaded.
