# Supplementary material for: Characterization of Two Small Heat Shock Protein Genes (Hsp17.4 and Hs20.3) from Sitodiplosis mosellana, and Their Expression Regulation during Diapause
Source: Insects. 2021 Jan 29;12(2):119. doi: 10.3390/insects12020119 (PMC7911813; doi:10.3390/insects12020119)
Supplement: Supplementary file 1 [file insects-12-00119-s001.pdf]

SmHsp17.4

```
AGTCGAATCTAAAGCATTCCAAAGTTGAAACGCGAACCGTTTTCGTTGAATTAATCCAGCAGACTCAAGTGAAAATTCATTCCAAAGTTTAAGTGATTTGAAATAAGAGAACAATTTAAG   120
AGATACTTTTGTGTCGTCAGAAAAATGTCGTTGATTCCATTCCTCTTGGAAGATCTCTATCCAACCAGCCATCATCGATTCGGAACTGGTTATTACCCGCGTCATTTGTGGAAAAATTAC   240
                          M  S  L  I  P  F  L  L  E  D  L  Y  P  T  S  H  H  R  F  G  T  G  Y  Y  P  R  H  L  W  K  N  Y    32
GAAGGAATTGAGAAGAAATCCCACATTGGCAAGGATGGTTTCGAAGCGTCACTTGACGTTGGACACTTTCAACCCAACGAAATCACCGTGAAAACGGATCATCATTCGATTGTGATTCAT   360
 E  G  I  E  K  K  S  H  I  G  K  D  G  F  E  A  S  L  D  V  G  H  F  Q  P  N  E  I  T  V  K  T  D  H  H  S  I  V  I  H    72
GCCAAACACGAAGAGAAACAAGACGAGCACGGTTACATTTCGCGTGAATTTACACGTCGCTATGATTTACCCGAAGGCTTCAAGCCGGAGGATTTAATATCTACTCTCTCATCTGATGGT   480
 A  K  H  E  E  K  Q  D  E  H  G  Y  I  S  R  E  F  T  R  R  Y  D  L  P  E  G  F  K  P  E  D  L  I  S  T  L  S  S  D  G   112
GTTCTCTCGATTAAATGCCCAAAACATCAGGGATCAAATGTACGCCACGTTCAAATCCAACAAACGGGCCCAGTCAAACAGAGCATCAAGAGCAACGAGGAGAAAAAAGAGGGAAAGTAA   600
 V  L  S  I  K  C  P  K  H  Q  G  S  N  V  R  H  V  Q  I  Q  Q  T  G  P  V  K  Q  S  I  K  S  N  E  E  K  K  E  G  K  *   151
ACAATTACAAGCGCTTCTTATGAATTACCTACCTCAATCTATGATGGATTTTCACATAAATTCTATATCCATTTATCCATACATTTCGATTCATTTTTACGCTCTAATTGTTGTGTCTTA   720
TTCAGAAATCAGTAAATTTCAATCAATATAAAGGACCAAAAAAAAAAAAAAAA                                                                     773
```

SmHsp20.3

```
TTATACGAATCGTTAACGAAACGCCATTATTTCCTCTCAAATTTCAAAGTGTAAAGTGAAATTAAAGTAAACTTAGAAAAAATGTCTCTATTGCCACACATTTTTGACGACGTTCATCCA   120
                                                                                 M  S  L  L  P  H  I  F  D  D  V  H  P    13
TCTTGCAATCGTCCAGGCTGCCATCGTTCACCATTATCGCTTGACTGGTGGTTCAAACCAACCGATTATCCAGCTTTGCGATCGGCATTAGGCAGTCCTGCCACTATTTTGAAACAACAT   240
 S  C  N  R  P  G  C  H  R  S  P  L  S  L  D  W  W  F  K  P  T  D  Y  P  A  L  R  S  A  L  G  S  P  A  T  I  L  K  Q  H    53
TTCCCCGATTTAGCAAATTGGAACATGGAGCCAAAATGGGCAAGGACGGCTTTCAAGTGTGTGTTGATGTTCAACATTTTCTACCCAAAGAAATTGAAGTGAAAACTGAGAATAACACC   360
 F  P  D  F  S  K  L  E  H  G  A  K  M  G  K  D  G  F  Q  V  C  V  D  V  Q  H  F  L  P  K  E  I  E  V  K  T  E  N  N  T    93
GTTATCGTTCATGCAAAGCACGAGGAAAAAGAAGATGATCATGGTTACATTTCACGCGAATTCACCCGACGCTATACGCTGCCACAAGAATTCAAGATTGAAGATGTAACATCAACTCTT   480
 V  I  V  H  A  K  H  E  E  K  E  D  D  H  G  Y  I  S  R  E  F  T  R  R  Y  T  L  P  Q  E  F  K  I  E  D  V  T  S  T  L   133
TCATCGGATGGCATTTTAACGATCACAGCACCATCACCAACACCGGCCGTTGAAGGCAAAGTTCACCATATTCCAATCCAACAAACGGGACCAGCTCACTTGAATGTCGCCAGCAACGAA   600
 S  S  D  G  I  L  T  I  T  A  P  S  P  T  P  A  V  E  G  K  V  H  H  I  P  I  Q  Q  T  G  P  A  H  L  N  V  A  S  N  E   173
GAAGTGAAAGACGAAGAAAAATAATTATAATTTGTTTATGTGAAGAAAGAAAGTGCATTTAATGGTTCCAAAAAGTGAGGAAGATCTGGATTTTTCTGAATTTCGATGATCTAAGTCTAT   720
 E  V  K  D  E  E  K  *                                                                                                    180
ACTTATTTATTGAGTTTTCTATTTGATAAAATGTGATAAAAACTAAGAGCGAATTTTGAAATTCATTAAAAAAAAAAAAAAAA                                        803
```

Figure S1. Nucleotide and deduced amino acid sequences of SmHsp17.4 and SmHsp20.3. Start codons (ATG) and stop codons (TAA) are boxed. The α-crystallin domain was shaded. I/VXI/V motif was indicated with oval.

SmHsp17.4

```
AGTCGAATCTAAAGCATTCCAAAGTTGAAACGCGAACCGTTTTCGTTGAATTAATCCAGCAGACTCAAGTGAAAATTCATTCCAAAGTTTAAGTGATTTGAAATAAGAGAACAATTTAAG   120
AGATACTTTTGTGTCGTCAGAAAAATGTCGTTGATTCCATTCCTCTTGGAAGATCTCTATCCAACCAGCCATCATCGATTCGGAACTGGTTATTACCCGCGTCATTTGTGGAAAAATTAC   240
GAAGGAATTGAGAAGGTACTTTATTGAGTTACACAGTTGTGAAGTTAACTTTTGTTGACTAATCGGTACATAATCAATCTGTTGCAACACTAAAATCAAGAAATCCCACATTGGCAAGGA   360
TGGTTTCGAAGCGTCACTTGACGTTGGACACTTTCAACCCAACGAAATCACCGTGAAAACGGATCATCATTCGATTGTGATTCATGCCAAACACGAAGAGAAACAAGACGAGCACGGTTA   480
CATTTCGCGTGAATTTACACGTCGCTATGATTTACCCGAAGGCTTCAAGCCGGAGGATTTAATATCTACTCTCTCATCTGATGGTGTTCTCTCGATTAAATGCCCAAAACATCAGGGATC   600
AAATGTACGCCACGTTCAAATCCAACAAACGGGCCCAGTCAAACAGAGCATCAAGAGCAACGAGGAGAAAAAAGAGGGAAAGTAAACAATTACAAGCGCTTCTTATGAATTACCTACCTC   720
AATCTATGATGGATTTTCACATAAATTCTATATCCATTTATCCATACATTTCGATTCATTTTTACGCTCTAATTGTTGTGTCTTATTCAGAAATCAGTAAATTTCAATCAATATAAAGGA   840
CC                                                                                                                         842
```

Figure S2. Genomic DNA sequence of SmHsp17.4. Start codon (ATG) and stop codon (TAA) were boxed. The intron was shaded.
